# The frontier and perspective for tokamak development

**DOI:** 10.1093/nsr/nwz029

**Published:** 2019-03-16

**Authors:** Jiangang Li, Mingjiu Ni, Yu Lu

**Affiliations:** 1 Institute of Plasma Physics, Chinese Academy of Sciences, China; 2 University of Chinese Academy of Sciences, China; 3 Bureau of Frontier Sciences and Education, Chinese Academy of Sciences, China

Fusion is an ideal clean energy for human beings in the future. Significant progress has been achieved on tokamak research after successful construction of many large tokamak facilities and many years of experiments, which have established a sound base for tokamak experimental reactors. More than 10 MW fusion power has been produced in the Tokamak Fusion Testing Reactor (TFTR) [1] and Joint European Torus (JET) [2] tokamaks. An equivalent *Q* (*Q* = output fusion power/input external heating power) of over unity was reached in the JT-60U [3] device during the last century. Since 2000, world tokamak research has moved to the new generation of fully superconducting tokamaks for steady-state high-performance operation. The Experimental Advanced Superconducting Tokamak (EAST) [4] and Korean Superconducting Tokamak Advanced Research (KSTAR) [5] tokamaks have been built and very good progress has been made. Tokamak research has reached a new era with joint efforts from China, Europe, India, Japan, Korea, Russia and the USA to build the International Thermonuclear Experimental Reactor (ITER) [6]. ITER will bring tokamak fusion research to a state where burning plasma, in which alpha particle self-heating will be dominant, can be explored. Over the coming 10 years, the world fusion community will make joint efforts to finish the ITER construction and start scientific experiments. Meanwhile, most of the ITER party has its own strategic plan to approach the Demonstration (DEMO) fusion reactor and efforts have been made in this direction.

The world tokamak community has confidence that ITER will be successfully constructed without obstruction; the target for this is 2025. The scientific goal of ITER is to demonstrate the feasibility of 500 MW fusion power with *Q* = 10 lasting for 400 seconds. There are still some risks in meeting this goal. The frontier and the most important issues for the next 10 years are the control of off-normal events, confinement and transport physics of the burning plasma, high-power particle and heat flux exhaust on the divertor, energetic particle behavior, high-performance long pulse and steady-state operation, and tritium breeding and retention.

Transient events can release plasma energy in a very short time and could damage plasma-facing components easily. These phenomena include minor and major disruptions and very localized plasma edge instabilities. Any major disruption in ITER could easily destroy some of the plasma-facing components, and therefore it is essential to develop robust ways to avoid or mitigate disruptions to prevent plasma-facing material from being damaged, to allow achievement of high-performance plasma and to ensure continuous operation in ITER. An elevated effort is needed in theory, modeling and technology for more advanced control to achieve a reliable, disruption-free operation scenario with 99% reliability.

In ITER, a burning plasma will be tested while the plasma is heated by fusion-born alpha particles. Energetic alpha particles from fusion reactions heat and sustain the burning plasma, but they also bring plasma instabilities. It is clear that a significant loss of alpha/fast ions may degrade the plasma heating and current drive efficiency and may lead to significant loss of plasma performance. Theory and modeling predictions of alpha particle control and reliable alpha particle diagnostics are needed to be explored with simple technical solutions in preparation for the ITER burning plasma physics experiments in the near future.

High-performance steady-state H-mode operation with a fusion power of 500 MW over 400 s is the premier goal of ITER operation, which needs integrated control for many physical quantities from both the core and edge. To maintain high core plasma performance, efficient plasma heating and current drive in the H-mode scenario, high bootstrap current fraction and low impurity concentration are required simultaneously. In the edge and scrape-off layer regions, it is essential to have a controllable plasma surface interaction by using strong gas puffing to keep a low ion temperature to sustain low impurity generation and physical sputtering on the tungsten divertor. The current superconducting tokamak devices such as EAST and KSTAR should make continued effort on these problems and achieve steady-state high-performance plasmas.

In ITER, over 10 MW/m^2^ peak heat fluxes that nearly reach the present technological limit are foreseen and the ITER mono-block divertor solution is not sufficient for DEMO. The future DEMO divertor working conditions are very challenging and need both new physical (detached plasma and new divertor configuration) and technical (over 20 MW/m^2^ target heat load) solutions. Moreover, integrated experiment validation with a heat load between 10 to 20 MW/m^2^ on a long-pulse tokamak is also needed in the near future.

There are still many scientific and technical difficulties for successful construction and operation of the future DEMO after ITER in terms of plasma performance, enabling technologies, material and component performance and safety issues. At present, China is leading the efforts towards a tokamak DEMO, and the development roadmap of Chinese tokamak fusion is shown in Fig. 1. ITER technologies provide the foundation for the roadmap. Based on the accomplishment of the last 50 years, by participating, sharing and assimilating the techniques in ITER and fast domestic tokamak development, China can start its next step program, called the Chinese Fusion Engineering Testing Reactor (CFETR), which is a combination of engineering testing and DEMO validation. A higher magnetic field (6.5 T) was chosen compared with ITER (5.3 T). A higher magnetic field is fa-vored for better confinement and much lower transport. Using a higher magnetic field is the future approach for the DEMO reactor even though it will cause engineering difficulties, since fusion performance is proportional to the third order of the magnetic field. Steady-state operation and tritium breeding will be concentrated with relative low fusion power, which eliminates the difficulty of high-*Q* burning plasma operation and fusion material in its early phase of operation. Almost all DEMO-reactor-related issues will be tested in the late phase of experimental operation together with experiences from ITER *Q* = 10 operation results. The DEMO-like advanced and reliable steady-state operation scenario will be achieved by a combination of high bootstrap current and efficient auxiliary driven current. The goal of tritium self-sufficiency will be obtained with the techniques of high fuel burning rate (over 3%), advanced tritium breeding blankets and a large-scale tritium factory, together with an advanced safety operation for the burning plasma and material technology development. By conducting engineering test and DEMO validation experiments respectively on CFETR, tremendous leaps from the engineering test reactor to the DEMO reactor and eventually the prototype of a power plant will be achieved by the mid-2050s.

**Figure 1.**
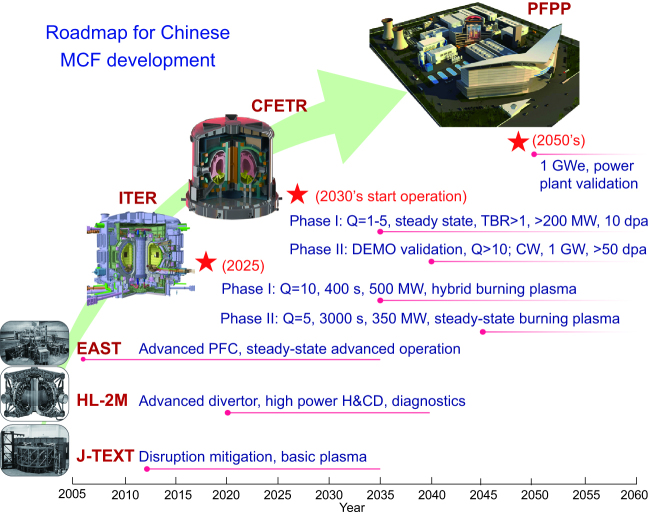
Roadmap for Chinese magnetic confined fusion (MCF) development.

Large-scale R&D that addresses the major challenges for CFETR should start as soon as possible, such as high-performance superconducting magnets including high-*T*_c_ magnets, advanced new Nb_3_Sn magnets, megawatt continuous wave gyrotron development, a steady-state negative neutral beam injection system, a remote handling (RH) system, a new DEMO divertor, advanced fusion materials and breeding blankets. Several testing facilities are needed for simulating future CFTER operation without a nuclear environment, such as a superconducting testing facility, a vacuum vessel for installation and removal by RH, a tritium exhaust testing facility, and a DT neutron system for blanket testing. With successful construction of these R&D and testing facilities, together with further engineering testing, large-scale simulation and modeling and tokamak experiments, a more solid basis will be established for the beginning of CFETR construction.

## References

[1] Hawryluk RJ , Batha S, Blanchard W et al Rev Mod Phys 1998; 70: 537–87.

[2] Keilhacker M , Watkins ML, the JET Team. Nucl Fusion 1999; 39: 209–34.

[3] Oyama N , Isayama A, Matsunaga G et al. Nucl Fusion 2009; 49: 065026.

[4] Wan Y , Li J, Weng P et al. Plasma Sci Technol 2006; 8: 253–4.

[5] Lee GS , Kim J, Hwang SM et al. Nucl Fusion 2000; 40: 575–82.

[6] ITER Physics . Nucl Fusion 1999; 39: 2137–74.

